# The Scientific Case for Animal Models: A Perspective From Musculoskeletal Researchers

**DOI:** 10.1096/fba.2025-00313

**Published:** 2026-02-09

**Authors:** Michael Hadjiargyrou, Blake E. Hildreth, Frank Ko, Uma Sankar, Tao Yang

**Affiliations:** ^1^ Department of Biological and Chemical Sciences New York Institute of Technology Old Westbury New York USA; ^2^ Department of Pathology, Heersink School of Medicine University of Alabama at Birmingham Birmingham Alabama USA; ^3^ Department of Anatomy and Cell Biology Rush University Medical Center Chicago Illinois USA; ^4^ Department of Anatomy, Cell Biology and Physiology Indiana University School of Medicine Indianapolis Indiana USA; ^5^ Department of Cell Biology Van Andel Institute Grand Rapids Michigan USA

**Keywords:** animal research, bone, bone cancer, fracture healing, Inherited Skeletal Disorders, musculoskeletal, New Approach Methodologies, osteoarthritis, osteoporosis

## Abstract

The National Institutes of Health (NIH) has launched a major initiative to expand human‐based New Approach Methodologies (NAMs) in biomedical research and reduce reliance on animal models. While NAMs offer powerful complementary tools, animal‐based research remains indispensable in musculoskeletal science for understanding complex cellular and systemic processes, disease onset and progression, and developing effective therapies. Foundational knowledge of embryonic development, disease mechanisms, tissue regeneration, gene function, and systemic pharmacology has emerged from animal models and will continue to do so. This review underscores the essential role of animal models in five key areas of musculoskeletal biology: osteoporosis, osteoarthritis, bone fracture repair and regeneration, bone cancer, and Inherited Skeletal Disorders (ISDs). We also examine NAMs including organoids, engineered scaffolds, organ‐on‐chip platforms, and Artificial Intelligence (AI)/computational modeling, highlighting their strengths in mechanistic and high‐throughput studies but also their limitations in replicating in vivo structural, physiological, biomechanical, and systemic complexity. Animal models remain the gold standard for exploring disease mechanisms, testing preclinical therapeutic and diagnostic efficacy and safety, and translating discoveries into clinical practice. Rather than replacing animal research, NAMs should be integrated as complementary approaches to advance understanding and innovation. Curtailing animal research would jeopardize medical progress and hinder life‐saving interventions for humans and animals alike. This review aims to inform the public and policymakers on the continued necessity of ethically conducted animal research as a cornerstone of musculoskeletal health.

## 1 Introduction

On April 29th, 2025, the National Institutes of Health (NIH) announced a new initiative to “expand innovative, human‐based science while reducing animal use in research” [1]. Further, the NIH Director, Dr. Jay Bhattacharya, stated that “For decades, our biomedical research system has relied heavily on animal models. With this initiative, NIH is ushering in a new era of innovation” [1]. While we agree that as scientists we should strive to “expand human‐based science”, it is important to recognize that not all research is clinically based. Non‐human based studies are equally significant and valuable. In fact, many fundamental discoveries that have advanced human health stemmed from basic science research using animal models. According to the Foundation for Biomedical Research, breakthroughs in the treatment of numerous human diseases, including Alzheimer's, cancer, cystic fibrosis, diabetes, and epilepsy were advanced through animal research [2]. Similarly, the Federation of American Societies for Experimental Biology (FASEB) in its Statement of Principles for the Use of Animals in Research and Education, “affirms the essential contribution of animals in research and education aimed at improving the health of both humans and animals”. And it further states that “the role of animals remains critical in understanding the fundamental processes of life and in developing treatments for injury and disease” [3]. We wholeheartedly agree!

The critical value of animal research lies in its ability to enhance our “*understanding* of *fundamental biological processes”* [3]. Examples of such fundamental processes include embryonic development—the formation of tissues, organs, and systems; onset and progression of diseases; pathophysiology of diseases; evaluation of pharmaceutical drugs and implantable devices; wound healing and tissue regeneration, etc. Moreover, the advent of genetic engineering has revolutionized our understanding of gene function and the contribution of specific cell types in tissue and organ development. This knowledge would not exist without specific transgenic animal models, predominantly in rodents, fish, frogs, flies, and worms. In our field of musculoskeletal biology, animal research has been pivotal to our understanding of musculoskeletal (bone, cartilage, tendons, ligaments, muscle) development and regeneration, bone remodeling, the influence of mechanical forces on bone density, and the mechanisms of bone and cartilage aging.

Similarly, animal research has been indispensable in developing treatments for skeletal disorders because these conditions involve complex, systemic interactions that cannot be replicated in non‐animal models. Osteoporosis therapies such as bisphosphonates, denosumab, and anabolic agents (e.g., teriparatide) were discovered and validated through in vivo studies that captured hormonal regulation, bone remodeling dynamics, and whole‐organism pharmacokinetics; features absent in cell cultures or organoids [4]. Similarly, therapeutics for rheumatoid arthritis, gout, and psoriatic arthritis required animal models to assess immune–joint–skeletal cross‐talk and systemic inflammation [5, 6, 7]. Disorders like fibrodysplasia ossificans progressiva and ankylosing spondylitis involve multi‐tissue ossification and immune signaling, which cannot be modeled in vitro because they depend on vascularization, endocrine feedback, and biomechanical loading [8, 9]. Non‐animal systems lack these integrated networks and cannot predict drug efficacy or safety under physiological conditions, making animal models uniquely capable of driving breakthroughs that have transformed clinical care.

While animal‐based research remains the gold standard for investigating complex biological and disease mechanisms, new technological advances such as in vitro 3D organoids, lab/organ‐on‐a‐chip, and *in silico* modeling offer valuable complementary approaches. However, these methods cannot replicate the full complexities of living organisms. Animal models provide distinct advantages, including anatomical, physiological, and biochemical similarities to humans; the ability to study whole‐body effects; disease onset and progression; and systemic drug metabolism [10]. Moreover, animal models continue to play a crucial role in biomedical research by advancing our understanding of many human and animal diseases as well as uncovering fundamental biological processes. Undoubtedly, the contribution of animal models will be indispensable to the continued development of new treatments and cures through gene therapy [11] and tissue engineering/regenerative medicine [12], vaccine development for emerging diseases [13], and advances in personalized medicine [14]. Importantly, animal research also supports the development of better treatments for veterinary care, benefiting farm, zoo, and wild animals as well as companion pets.

The study of musculoskeletal biology requires the consideration of systemic physiology, which represents the integrated function of multiple organ systems. This is because bone, muscle, and cartilage operate within a network of endocrine, vascular, immune, and mechanical interactions. Non‐animal systems such as cell cultures or organoids cannot replicate these whole‐body dynamics, including hormonal regulation by parathyroid hormone (PTH), calcitonin and vitamin D, vascular and neural input, and multi‐organ feedback loops that maintain mineral homeostasis. An important example is skeletal calcium, which exists in dynamic equilibrium with blood calcium to support vital processes like neural signal transmission and muscle contraction. Bone acts as a buffer, releasing or storing calcium in response to systemic cues through a process involving PTH, bone cells, kidney, and the gut. In vivo animal studies demonstrate the inextricable link between the rapid, cell‐mediated calcium flux in bone and hormone‐driven modulation of bone remodeling as well as the complex endocrine and metabolic feedback of systemic calcium during dietary calcium fluctuations, immobilization, pregnancy and lactation, and steroid‐induced osteopenia, closely mimicking human physiology [15, 16, 17, 18, 19, 20]. Non‐animal systems such as in vitro and computational models lack the integrated network of bone‐endocrine‐vascular‐immune interactions and rapid feedback dynamics necessary to model skeletal contributions to systemic calcium equilibrium.

Further, bone and joint cells do not act in isolation, as their intracellular signaling is influenced by endocrine hormones, neural inputs, vascular supply, immune mediators, and mechanical signals, creating feedback loops that regulate remodeling and mineral homeostasis. For example, the activities of osteoblasts and osteoclasts are modulated by systemic factors such as PTH, calcitonin, and inflammatory cytokines, which act across multiple organs [21, 22]. These interactions alter intracellular signaling pathways, gene expression, and metabolic states in ways that cannot be reproduced in cell culture or organoid models. Non‐animal models lack integrated circulation, hormonal gradients, and biomechanical loading, making them incapable of replicating the dynamic systemic environment that shapes cellular behavior in vivo.

**FIGURE 1:**
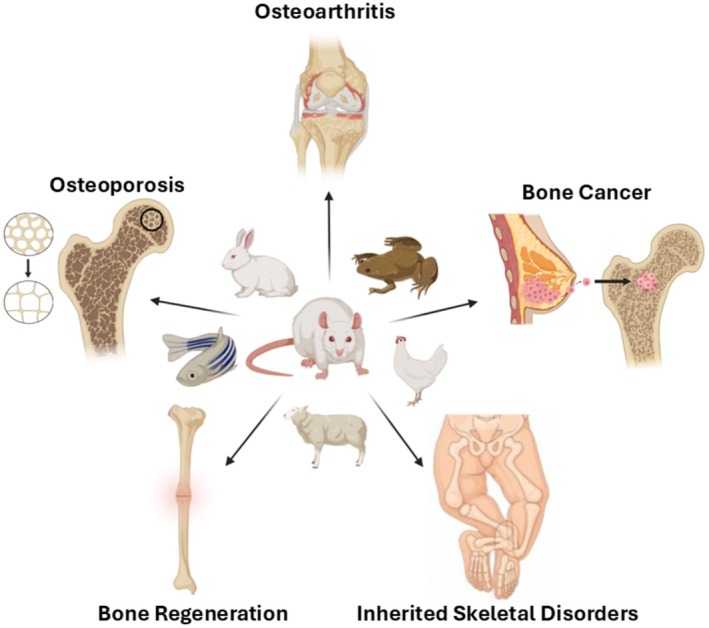
Schematic illustrating the contribution of animal models to mechanistic and translational research in musculoskeletal diseases. Figure was created using BioRender.

In this perspective, we have structured the manuscript in a series of short vignettes, each examining the scientific value and limitations of an animal model alongside New Approach Methodologies (NAMs) designed to reduce or replace animal use in research. As musculoskeletal researchers, we specifically focus our vignettes on animal models for osteoporosis, osteoarthritis, bone cancer, fracture repair/regeneration, and rare bone diseases (Figure 1). We hope that this concise review, written in mind for public consumption, can be used as a springboard to engage and educate the public on the necessity of animal research and its direct impact on human and animal health and lifespan. Additionally, we hope this manuscript underscores to our Congressional Representatives and Senators, who are responsible for policy making and research funding, the critical necessity of sustaining animal‐based research. Abandoning or banning such research, as advocated by certain vocal segments of our society, would not only hamper future breakthroughs in medicine but also cost human and animal lives.

## 2 Understanding Osteoporosis Through Animal Models

One of the devastating consequences of aging is its effects on the skeleton, predominantly in the form of osteopenia, characterized by a decrease in bone mineral density (BMD) below normal reference values [23]. Osteopenia involves the uncoupling of osteoclast–osteoblast activity, which results in a quantitative bone mass decrease. If left untreated, osteopenia leads to osteoporosis, a disease defined by progressive bone loss and skeletal deterioration leading to bone fragility and eventually fractures [24]. The World Health Organization (WHO) defines osteoporosis as a “progressive systemic skeletal disease characterized by low bone mass and microarchitectural deterioration of bone tissue, with a consequent increase in bone fragility and susceptibility to fracture” [25]. This deterioration in bone density results in brittle, fragile bones, predominantly in the hip, spine, and wrist, that fracture easily [26]. According to the American Society for Bone and Mineral Research, “osteoporosis is a major public health threat for 44 million Americans” and “of the 10 million who have osteoporosis, 80 percent are women and another 34 million have low bone mass and are at risk”. In addition, “2 million men have osteoporosis and about 12 million more are at risk” [27]. Understanding this devastating and systemic disease that affects millions of people in the United States and worldwide requires animal models that accurately capture the uncoupling of osteoclast–osteoblast activity as well as the resulting decrease in bone BMD decrease that leads to osteoporosis.

While in vitro systems can partially elucidate cellular mechanisms, they fail to address the systemic aspects of the disease or the associated loss of tissue—factors that can only be addressed at the organismic level. Recent advances in microfluidics, organoids and organ‐on‐chip approaches offer promising tools to complement osteoporosis research. However, these approaches exhibit significant limitations and challenges such as: (1) the development of a blood mimetic medium that provides the nutrients, hormones, and growth factors required for the maintenance of the various bone cell types; (2) standardization of the manufacturing process in the absence of commonly accepted protocols and materials for creating these devices; (3) overcoming limitations with respect to organ sizes, transportation rates between multiple tissues/organs, and liquid‐to‐cell ratio, all required to mimic physiologically relevant conditions; and more importantly, (4) establishing experimental conditions that truly approximate in vivo physiology [28] (Table 1).

**TABLE 1:** Animal models versus NAMs in musculoskeletal research.

Overall	Advantages	Disadvantages
Animal models	Ability to capture systemic interactions, and the complexity of multi‐tissue and multi‐organ communications. Enable longitudinal studies of disease progression. Allow for the evaluation of musculoskeletal pain behavior. Established models exist for multiple musculoskeletal conditions. Ability to validate therapeutic targets in vivo, in a physiologically relevant system. Provide clinically relevant data for drug testing, safety, PK/PD, and efficacy. Allow integration with NAMs for hybrid approaches.	Species‐specific differences reduce translational accuracy. Time‐consuming and costly to induce disease or maintain models. Surgical procedures and animal suffering involved.
NAMs	Faster and cost‐effective for mechanistic studies. High‐throughput capabilities for drug screening and biomaterial testing. Allows precise control of experimental conditions and standardization, which enhances reproducibility of findings. Human‐relevant cellular/genomic contexts. Reduces animal use and ethical concerns. Fast and cost‐effective.	Does not capture the in vivo complexity of multi‐tissue, multi‐organ cross‐talk and systemic influences on the disease. Limited capacity to model chronic and localized pain. Does not recapitulate metabolic and immune responses as well as their impact on the musculoskeletal system. Organ size limitations Require validation (often in animal models). Technical challenges and standardization may be lacking. Time consuming to standardize.
Osteoarthritis		
Animal models	Capture whole‐joint complexity Assessment of longitudinal pain behavior associated with OA, and the effect of drugs on OA pain. Allow longitudinal assessment of osteoarthritis pathogenesis. Assessment of therapy effects on disease prevention and disease progression in OA.	Species‐specific differences in joint mechanics and cartilage thickness alters osteoarthritis progression. Limited predictive power for human drug metabolism.
NAMs	Innovative platforms (3D cultures, joint‐on‐chip)	Cannot model whole joint complexity and systemic influences on OA. Cannot model joint pain.
Osteoporosis		
Animal models	Can model the complexity of osteoporosis and osteopenia Well established genetic and induced models of osteoporosis and osteopenia exist. Capture multi‐organ interactions and systemic effects on bone biology.	Requirement of surgical procedures to induce disease – ovariectomy, orchiectomy and disuse models. Results from osteoporotic animal models may not translate exactly to human disease.
NAMs	Simulate bone tissue structure and spatial morphology. Mimic osteoblast/osteoclast communication in 2D and 3D models.	Cannot replicate disease complexity. Cannot address challenges with the systemic aspect of osteoporosis. Bone organoids do not recapitulate complete organ structures and functions.
Bone Fracture Healing		
Animal models	Capture systemic, multi‐tissue and multicellular responses to skeletal trauma and bone regeneration. Established models available to replicate clinical scenarios in orthopaedic or dental implant placement procedures. Genetic models available to probe mechanisms regulating bone healing. Allow studying therapeutic efficacy in promoting bone healing and off‐target effects.	Species variation in response to skeletal trauma and bone healing Experimental confounders such as animal handlers, surgeons, housing or analgesia/anesthesia.
NAMs	Allow mechanistic studies using cells and tissue derived from human fracture site or callus. High mechanistic resolution by elucidating bone regenerative pathway at a cellular level	Cannot replicate multi‐cell and multi‐tissue responses to skeletal trauma, which are crucial for bone regeneration.
Inherited Skeletal Diseases (ISD)		
Animal models	Counterpart mutations substantially recapitulate human ISD phenotypes. The well‐established and rich resources of genetically engineered mouse models facilitate ISD mechanistic study	Variation in the severity of ISD phenotypes between humans and mice.
NAMs	Have direct clinical relevance in modeling human ISDs. Allow the direct study of ISDs in human cellular and genomic contexts. Enable efficient ISD material generation and high‐throughput drug screening.	The small and simplified iPSC‐derived organoids have limitations for downstream functional analyses and treatment evaluations.
Bone Cancer		
Animal models	Allow clinically relevant investigation of bone tumors and bone metastasis of solid tumors in a physiological context. Capture the contribution of the immune system to cancer progression in bone. Enable the development of patient‐specific therapies through xenograft models of patient‐derived cancer cells. Allow the assessment of tumor‐induced bone pain. Enable mechanistic studies of primary bone cancer as well as bone metastasis and secondary metastatic spread of bone‐lodged cancer cells	Limited availability of humanized models to study cancer in the bone. Xenotransplantation models lacking an intact immune system reduce translation potential.
NAMs	Model direct interactions between tumor cells and bone cells. Allow the study of cancer cell‐bone cell and cancer cell‐bone extracellular matrix interactions through engineered scaffolds. Model in vivo tumor‐bone niche complexity through rapid prototyping using 3D/4D scaffolds.	Cannot model direct interactions between tumor cells, cells in bone, and the extracellular matrix

Abbreviations: ISD, Inherited Skeletal Diseases; NAMS, New Approach Methodologies; OS, Osteoarthritis; PK/PD, Pharmacokinetics/Pharmacodynamics.

Similarly, mathematical modeling and artificial intelligence (AI) approaches to osteoporosis research have been implemented to better understand mechanisms of bone remodeling, predict disease progression, and evaluate treatment strategies. However, these efforts have yielded only limited success. Many studies using mathematical modeling and AI fall into the realm of drug regimens for the treatment of osteoporosis [29]. The management of osteoporosis is another area of research utilizing these approaches [30]. Additionally, computer simulation of bone remodeling has been reported [31]. The main challenges associated with mathematical modeling and AI are: (1) validating models with robust clinical and experimental data; (2) accounting for individual variability (age, genetics, comorbidities, medication history); and (3) representing or mimicking the complexity of the disease [32] (Table 1).

In contrast to the limitations of in vitro and mathematical approaches, research using specific animal models have generated a wealth of data that cover different aspects of osteoporosis, including disease onset and progression; molecular and cellular mechanisms; pathophysiology; and both pharmacological and non‐pharmacological interventions. In fact, a PubMed search using the terms “osteoporosis animal models”, yields over 4700 manuscripts, underscoring their widespread use in osteoporosis research. Specific examples of animal models for osteoporosis include ovariectomized (OVX) mice and rats, aged mice and rats, normal and OVX sheep, primates, dogs, and genetically modified mice. Animal models remain the gold standard in osteoporosis research, offering distinct advantages such as replicating natural age‐related bone loss, providing reproducible results (rodents), sharing similar bone size and architecture with humans (large animal models), exhibiting the closest bone biology/physiology to humans (primates) and enabling precise genetic control and complete mechanistic studies (genetically modified mice) [33, 34, 35, 36, 37, 38]. More importantly, animal model‐based research has led to the discovery of a number of pharmaceutical agents that are currently used in the clinic to treat osteoporosis, including Teriparatide, Abaloparatide, Romosozumab and Zoledronate [39]. Collectively, these drugs represent major achievements in osteoporosis research that ultimately benefits humanity. More importantly, none of them would have reached the clinic without rigorous in vivo preclinical testing.

## 3 Understanding Osteoarthritis Through Animal Models

Osteoarthritis (OA) is a major cause of pain and disability worldwide, affecting over 595 million people globally and 32.5 million adults in the United States, and costing over $480 billion annually in treatment and lost productivity [40, 41, 42, 43, 44]. Though commonly associated with aging, sports‐related injuries such as ligament tears and joint fractures can lead to post‐traumatic OA (PTOA), accounting for ~12% of all OA cases [45]. OA is a complex, multifactorial disease impacting the entire joint, including articular cartilage, subchondral bone, synovium, ligaments, tendons, and muscle [46, 47, 48]. Pain management typically involves nonsteroidal anti‐inflammatory drugs (NSAIDs), analgesics, and opioids, despite limited efficacy in improving function [49]. Between 2007 and 2014, 17% of OA patients were prescribed opioids, representing 53% of all opioid prescriptions in the United States and contributing significantly to the national opioid crisis [50, 51, 52]. Currently, no FDA‐approved disease‐modifying OA drugs (DMOADs) exist, leaving pain management, lifestyle interventions (e.g., weight control and exercise), and end‐stage joint replacement surgery as the primary treatment options [45, 46].

A comprehensive understanding of the cellular and molecular mechanisms underlying the complex OA pathology and its multifactorial etiology is crucial for developing efficacious DMOADs that can slow disease progression and alleviate pain. Preclinical animal models are critical to this effort [53] and their foundational contributions to elucidating OA pathogenesis and their continued utility in identifying and validating therapeutic targets underscore their indispensable role in translational research.

From a historical perspective, by the early 1900s, physicians began to challenge the long‐standing view of OA as merely an age‐related “wear and tear” condition characterized by articular cartilage breakdown, drawing on clinical observations and animal studies [54, 55]. Around the turn of the twentieth century, pivotal research using surgically induced PTOA models in rabbits, dogs, guinea pigs, and mice provided compelling evidence that OA is a disease affecting the entire joint [53]. Subsequent investigations into spontaneous OA, both naturally occurring and genetically induced, in small and large animal models that closely mimic human pathology have been instrumental in uncovering the cellular, genetic, and molecular mechanisms underlying the disease and in guiding the development of novel therapeutic strategies [56, 57, 58, 59, 60, 61].

Further refinement of animal models, including both surgical and non‐invasive induction of PTOA in normal and genetically modified mice, has deepened our understanding of how joint anatomy and biomechanics contribute to OA pathogenesis. These models have also illuminated the roles of age, genetics, diet, physical activity, and biological sex—key risk factors shared with human OA—in influencing disease incidence and severity. Importantly, these studies laid the groundwork for recognizing distinct OA subtypes, species‐specific differences in disease manifestation, and the temporally regulated nature of pathogenesis driven by diverse genetic and molecular determinants [62, 63, 64, 65, 66, 67, 68, 69].

Joint swelling and pain are hallmarks of symptomatic OA. While healthy articular cartilage lacks innervation, other joint tissues such as subchondral bone, synovial membrane, meniscus, and ligaments are innervated by sensory neurons originating from the lumbar 3–5 dorsal root ganglia of the spinal cord [70]. Studies using animal models have demonstrated that inflammation can lead to neovascularization breaching the osteochondral junction, contributing to pain and disability in a sex‐dependent manner [71, 72]. Thus, animal models have been pivotal in capturing the complexity of cellular and tissue interactions within the joint, as well as systemic regulatory mechanisms that drive structural pathology and pain in OA.

The recent NIH initiative mandates the consideration of NAMs in OA research and includes 3D cultures, human tissue explants, and scaffold‐based systems that mimic aspects of joint architecture and allow mechanical stimulation. Joint‐on‐chip platforms simulate tissue crosstalk and loading, while *in silico* models predict OA signaling and drug responses [73, 74, 75]. NAMs also support high‐throughput drug screening and mechanistic studies, reducing ethical concerns of animal use. However, they are technically challenging and require validation in animal models. They also fail to replicate drug metabolism and off‐target effects, and AI‐based platforms rely heavily on animal‐derived data. Critically, current non‐animal models do not capture the complex, inter‐tissue communication in the joint or systemic factors that drive OA progression (Table 1).

Over the past two decades, animal models have significantly advanced our understanding of OA mechanisms and delineating disease subtypes in humans. These models have enabled researchers to investigate the complex interplay between cartilage degradation, subchondral bone remodeling, synovial inflammation, and pain pathways—features that are difficult to replicate in non‐animal simplified systems. While NAMs, including organ‐on‐chip platforms and computational approaches, represent promising research avenues for mechanistic studies and high‐throughput drug screening, they cannot fully reproduce the multifactorial structural pathology and symptomatic manifestations characteristic of OA. Further, animal models remain essential for validating therapeutic targets, assessing pharmacokinetics and pharmacodynamics, and evaluating safety and efficacy in a physiologically relevant context. Lastly, they provide critical insights into disease progression under mechanical loading and systemic influences, which are essential for translating preclinical findings into successful DMOAD development pipelines. Together, these factors highlight the critical role of animal models in closing the gap between fundamental research and clinical translation in OA.

## 4 Understanding Fracture Repair and Bone Regeneration Through Animal Models

Skeletal fractures, which can be caused by aging, excessive physical activity, high‐energy trauma, or diseases, are one of the leading causes of hospitalization from injuries [76, 77, 78]. Tibia, fibula, and ankle fractures lead to 569,000 hospitalization days, resulting in a significant loss of productivity for patients and increased cost of care [79]. When including both the direct cost of care and the loss of productivity, skeletal fractures can cost up to $31,000 per limb [80]. Approximately 5 to 10% of skeletal fractures also lead to incomplete healing such as non‐union, further complicating the recovery process for patients and increasing the cost of care [81, 82, 83, 84]. Given the incidence of orthopedic and dental procedures that are dependent on the success of fracture healing and bone regeneration, such as skeletal fractures (~500,000 in the United States annually), joint replacement surgeries (~900,000 in the United States annually), dental implant placement procedures (~2,000,000 in the United States annually) [85, 86, 87, 88], and treatment of large bone defect healing through allogenic or biomimetic tissue transplantation [89, 90, 91, 92], further understanding the mechanisms of fracture healing and bone regeneration could lead to novel therapeutic opportunities that can enhance the success of clinical procedures that affect a large number of people.

Fracture healing and bone regeneration typically occur via two distinct processes: intramembranous or endochondral ossification pathways. These two distinct pathways share many of the features observed during embryonic development of the long bones (endochondral) and skulls (intramembranous), such as angiogenesis, progenitor cell condensation, differentiation of progenitors to bone forming osteoblasts, and tissue remodeling [93, 94, 95, 96]. Important distinction remains between bone development and repair, such as initial hematoma formation and a spike in inflammatory signaling following skeletal trauma. Over the course of bone repair these distinct processes, hematoma, inflammation, angiogenesis, and bone formation, are carefully coordinated by heterogeneous cell types such as macrophages, marrow and periosteal stem cells, endothelial cells, and osteoblasts. Due to the complexity of this process, animal models of fracture healing and bone regeneration have been widely used to understand the underlying mechanisms and treatment options for skeletal trauma.

Animal models have been a powerful tool to replicate several different clinical scenarios observed in fracture healing and bone regeneration and test the efficacy of different treatment strategies. These models can mimic the bone repair processes such as stabilizing fractures with intramedullary rod [97, 98], fracture nonunion [99, 100], stress fractures [101, 102], complex skeletal trauma [103, 104], digit tip regeneration [105, 106], calvarial defect healing [107, 108], and much more. Rigorous studies of animal models have revealed intricate processes of the aforementioned bone repair processes and allowed testing of new pharmacological therapies and medical devices [109, 110, 111, 112, 113, 114, 115]. The knowledge gained from these studies has now allowed clinicians to employ bone morphogenetic protein for the treatment of fracture nonunion/spinal fusion and continuous passive motion for tibial fractures [116, 117], to name a few. Specifically, INFUSE Bone Graft containing bone morphogenetic protein‐2 has been FDA‐approved since 2004 for use in acute tibial fractures, where its application increased the rate of radiographic healing [118, 119].

However, ethical considerations cannot be ignored when using animal models of fracture repair and bone regeneration, especially considering the surgical or traumatic insults introduced to animals to replicate human fractures. As a result, prior to the commencement of studies that involve animal studies, a rigorous institutional ethical review is performed with consideration of animal wellbeing, 3R principles (replace, reduce, refine), and alternative models. Unfortunately, alternative models such as in vitro culture systems or computational models have been unable to replicate the complex physiological and biomechanical interactions that occur during fracture healing and bone regeneration [120]. While we have observed significant advancements in 3D osteogenic constructs and microfluidic organ‐on‐chip models [121, 122], these in vitro models have yet to incorporate the functional hematopoietic system and nerves [123, 124, 125, 126], hampering the study of the initial acute inflammatory phase and neural components that occur during bone repair. This highlights the continual need to employ animal models to study fracture repair and bone regeneration (Table 1).

## 5 Understanding Bone Cancer Through Animal Models

Primary bone tumors arise in bone and bone metastasis results from tumors spreading to bone. Both remain a therapeutic challenge, with prognosis depending on the specific tumor type. There is a significant reduction in patient quality of life and survival once primary bone tumors metastasize or spread to bone. In osteosarcoma, the most aggressive primary bone tumor, those with multi‐systemic metastasis at initial presentation have one‐ and five‐year survival rates of 54% and 9% [127]. In bone metastasis, one‐year survival is lowest in lung (10%) and highest in breast (51%) cancer; however, breast cancer drops to 10% after 5 years [128]. Bone is a complex organ, continuously influenced by local and systemic factors. From this aspect, animal models are central in investigating tumors arising from, or spreading to bone, to improve patient well‐being and survival.

The development of spontaneous primary bone tumors or bone metastasis is rare in laboratory animal models. In addition, spontaneous tumors in genetically engineered models are not as relevant since (1) primary bone tumors tend to arise in the axial, rather than appendicular, skeleton and can be polyostotic and (2) they rarely metastasize to bone [129, 130, 131]. Therefore, the development of genetic models that more faithfully mimic the spontaneous human disease regarding primary tumors or bone metastasis is needed. From this, establishing primary bone tumors and bone metastasis in vivo is achieved largely through injecting tumor cell lines into mice or rats. Cells are primarily from (1) the same species/strain (auto/allografts) or (2) humans in immunocompromised hosts (xenografts). Primary tumors involve implanting tumor cells/grafts into bone [132, 133, 134]. For bone metastasis, tumor cells are injected into the orthotopic site, vasculature, or bone [134, 135]. Locoregional intravascular injection increases bone metastasis incidence and study population uniformity, reducing animal numbers, which also applies with direct tumor cell or graft implantation into bone [134, 136, 137]. Of clinical relevance, cell line‐based syngeneic models are immunocompetent. Human xenograft models can employ patient‐derived xenografts (PDXs), enabling patient‐specific therapies [133, 134, 138, 139]. However, “humanized” models are currently limited to human tumor cells placed into immunocompromised rodents transplanted with only human bone/bone constructs [140, 141, 142]. Additional models involve subcutaneously implanting (1) bone cell population(s) with or without carriers (i.e., ossicles); (2) vertebrae (vossicles); or (3) bone discs/fragments [140, 142, 143, 144, 145, 146, 147]. These have unique benefits over skeletal tumors, including the ability to: (1) recreate the bone microenvironment; (2) avoid tumor‐induced bone pain; (3) be removed over time if multiple, reducing animal numbers; (4) inject tumor cells into them; (5) have tumor cells metastasize to them; and (6) create a highly uniform sample population. These animal models have great utility and standardization, allowing inter‐study comparison and reducing the numbers of animals used.

The historical and simplest NAM are in vitro co‐cultures, where one or more bone cell type(s) are exposed to tumor cells or their secreted proteins [148]. This has evolved to using (1) organoids and (2) rodent cranial or long bones or human bone fragments exposed to tumor cells or their secretory products [149, 150, 151, 152]. Engineered scaffolds more closely mimic bone, can be synthetic, natural, or hybrid in design, and allow for modulating cell type(s) and extracellular organic/inorganic components [153, 154, 155]. These yield infinite possibilities to (1) define elements' distinct contributions and (2) evaluate therapeutics, culture conditions, and biomechanics. Rapid prototyping now allows for 3D/4D scaffolds and highly complex and reproducible bone models [153, 154, 155]. In vitro systems can model species‐specific differences with a high degree of standardization. Rapid prototyping enables further scaffold optimization and reproducibility. However, NAMs are limited by the inability to completely recapitulate the complex cellular/structural network of bone and the impact of systemic factors (Table 1).

Enhanced bone turnover is implicated in bone metastasis [156]. Bone's sinusoidal vascular endothelium allows tumor cell extravasation [157, 158]. Bone‐centric mechanisms regulate tumor cell dormancy [159, 160]. These all require a complete bone microenvironment. Bone may be the first metastatic site, then spreading elsewhere, which cannot be modeled in vitro [161]. Evaluating hormonal influence is invaluable in (1) breast cancer, where estrogen deficiency‐induced bone loss promotes bone metastasis/outgrowth and (2) prostate cancer, since castration resistance drives disease progression [162, 163, 164]. Weight bearing in mice, as in humans, results in direct and indirect effects on tumor and/or bone cells [165]. Animals are also critical for (1) evaluating surgical methods and therapeutics and (2) developing diagnostic and “theranostic” modalities, requiring circulation for agent delivery. As such, animal‐based research in bone cancer is indispensable (Table 1).

## 6 Understanding Inherited Skeletal Disorders Through Animal Models

Inherited skeletal disorders (ISDs) are caused by monogenic or oligogenic mutations in the genes responsible for skeletal cell differentiation and homeostasis, matrix remodeling, or communication between the skeletal system and other organs. They form a large and complex group of disorders that cause morphological and functional deficits of the skeletal system; many of them are severe and inborn, syndromic, causing early death or permanent disability, and difficult to treat [166]. Besides, chronic skeletal disorders, such as osteoporosis and OA, have strong genetic associations and may share similar mechanisms with the ISDs to various degrees. For example, the discovery of a genetic variant in the *LRP5* gene causes either low bone mass (Osteoporosis‐Pseudoglioma Syndrome) or high bone mass (Van Buchem disease), revealing the crucial role of WNT signaling in bone homeostasis and inspiring treatments for osteoporosis [167, 168, 169]. In this sense, ISD research has a broad impact on human skeletal health.

Genetic mouse models are a unique and instrumental resource for studying ISDs. Coding genes are highly conserved among mammals; approximately 99% of human genes have counterparts in mice [170]. In 96% of cases, the mutations responsible for human skeletal diseases are faithfully and substantially recapitulated in mice carrying counterpart mutations [171]. Moreover, decades of efforts by mouse geneticists, developmental biologists, consortiums, and pharmaceutical companies have made available a vast pool of genetic mouse models. In brief, these mouse models are created through global or conditional (1) loss or mutation of genes implicated in specific disease states or (2) gain of function of disease‐related mutations. These largely rely on recombinase technology (Cre or Flp), and more recently CRISPR, to either (1) remove genetic sequences of interest or (2) excise integrated “stop” sequences upstream of an introduced mutation of interest which then allows for its expression. These technologies allow for great modularity in genetic modification—that is, throughout the lifespan of the animal or at distinct time points and either globally or in distinct cell types. Cre recombinases are available in almost all known skeletal cells and organs implicated in regulating bone [172]. The advent of genome editing technology, which has significantly increased the cost‐effectiveness and throughput of creating new genetic mouse models [173], has also facilitated a quick expansion of this resource.

Genetic mouse models provide an important in vivo context, enabling the assessment of molecular mechanisms of these mutations with precise spatial and temporal resolution. They offer a holistic view of disease development and treatment effects, uncovering previously unexplored syndromic aspects, and allow us to study the communication between bone and other organs during disease progression. Additionally, this resource confers great flexibility for genetic rescue, which has a crucial impact on exploring disease mechanisms and inspiring innovative treatment ideas. Moreover, genetic mouse models have inspired new discoveries in human ISD etiology and mechanisms, facilitating the development of effective treatments. For example, *CRTAP* mutations, which are a genetic etiology of recessive osteogenesis imperfecta (OI), were discovered through the study of a *Crtap* loss‐of‐function mouse model [174]. Additionally, the anti‐TGF‐β treatment for OI was initially inspired by the excessive TGF‐β signaling observed in mouse genetic models for OI [175].

The use of induced pluripotent stem cell (iPSC)‐derived bone organoids as an NAM for ISD research has recently emerged. A study published this year reported that bone organoids derived from the iPSCs of an osteogenesis imperfecta (OI) patient produced typical misfolded triple helical collagen fibril and exhibited ER stress [176]. It demonstrates the advantages of directly studying diseases in a human genetic context and of eliminating the burden of creating a genetic animal model for a corresponding human genetic mutation. However, ex vivo, miniaturized, and simplified culture models also have obvious limitations, which are deemed complementary to animal models. The cultured iPSC‐derived bone organoids are generally rather small and lack tissue and systemic context, which may impact both cell differentiation and extracellular matrix accumulation, organization, and signaling. These deficiencies likely affect the accuracy of recapitulating ISD pathology and restrict their use for follow‐up mechanical, structural, or functional testing, which are crucial for exploring the disease mechanisms and evaluating pathology and treatment outcomes. It is plausible that the above work utilized the transplantation of these iPSC‐derived bone organoids into the renal capsules of immunodeficient NSG (NOD scid gamma) mice, which enables the analysis for more disease‐relevant osteogenesis, mineralization, and bone cell morphology [176]. This is an exemplary case where the limitations of NAMs are overcome by combining them with animal models.

In summary, the significant strengths of these aforementioned transgenic models are currently irreplaceable by NAMs (Table 1). Therefore, future studies of ISDs should be effective‐oriented by focusing on complementing these two systems by integrating their unique advantages, rather than arbitrarily reducing the research of one of them. It is foreseeable that technology will continue to evolve in the creation and use of animal models, ensuring they remain an effective and indispensable part of ISD research.

## 7 Discussion

Our review highlights the necessity of animal models and the complementary role of NAMs in advancing our knowledge in musculoskeletal research and developing novel therapeutic opportunities to enhance the quality of life for patients suffering from musculoskeletal disorders. While in this review we have focused on animal models of osteoporosis, osteoarthritis, fracture healing, bone, and cancer, and rare bone diseases, other animal models of sarcopenia, ligament injuries, tendinopathy, etc., are just as important in musculoskeletal research [177, 178, 179]. These animal models, including the ones described in this review, enable researchers to study heterogeneous and complex musculoskeletal tissues that cannot be readily recapitulated by NAMs and provide opportunities to develop interventions and treatments for diseases that affect multiple musculoskeletal tissues such as OA, skeletal traumas, and osteosarcoma. In addition, the extensive availability of genetically engineered animal models has created tremendous opportunities to deepen our mechanistic understanding of musculoskeletal pathologies, insights that are not possible through the use of NAMs [180, 181, 182]. Although NAMs can be genetically modified, they lack the physiological context of whole organisms to model and study complex musculoskeletal diseases. Animal models will remain vital for hypothesis testing, validation of in vitro and NAMs, and preclinical safety and efficacy assessments. Moreover, AI and computational models require vast datasets derived from animal studies for effective training. In short, the future of musculoskeletal research is intrinsically linked to animal models, and to their thoughtful and vital integration with emerging technologies like NAMs.

While NAMs should be embraced where scientifically appropriate, their fundamental limitations in capturing the complexity of musculoskeletal systems and associated diseases must also be recognized (Table 1). Advances in NAMs such as organoids, organ‐on‐chip platforms, and computational models excel in mechanistic dissection and high‐throughput screening. However, they cannot replicate physiologically relevant inter‐tissue communication, systemic drug metabolism, or the biomechanical environment essential for understanding musculoskeletal disease progression and therapeutic efficacy. NAMs can, however, complement animal research by refining experimental parameters and reducing unnecessary replication.

Without continued animal research, conducted ethically and responsibly, progress toward developing new treatments for the millions of people worldwide suffering from arthritis, those at risk for osteoporosis, and countless others affected by bone injuries, cancer, and inherited disorders would halt. The goal should not be to choose between animal models and NAMs, but instead to integrate both strategically and wisely to save lives and improve musculoskeletal research and ultimately, health. This complementary approach ensures scientific rigor and ethical responsibility, aligning with the NIH's stated goals of expanding innovation while upholding humane practices. Finally, as our policy makers and funding agencies shape future research directions, it is imperative that they recognize the indispensable role of animal‐based research as a scientifically justified endeavor that safeguards future innovation and the development of treatments benefiting both human and animal health.

## Author Contributions

All authors were involved in drafting and revising the manuscript. All authors approve of the final manuscript.

## Conflicts of Interest

The authors declare no conflicts of interest.

## Data Availability

No new data. Data sharing not applicable to this article as no datasets were generated or analyzed during the current study.
